# Pneumatocele during sorafenib therapy: first report of an unusual complication

**DOI:** 10.18632/oncotarget.23599

**Published:** 2017-12-22

**Authors:** Paloma Sangro, Idoia Bilbao, Nerea Fernández-Ros, Mercedes Iñarrairaegui, Javier Zulueta, JI Bilbao, Bruno Sangro

**Affiliations:** ^1^ Liver Unit, Department of Internal Medicine, Clínica Universidad de Navarra-IDISNA, Pamplona, Navarra, Spain; ^2^ Liver Unit, Department of Internal Medicine, Clínica Universidad de Navarra-IDISNA and CIBEREHD, Pamplona, Navarra, Spain; ^3^ Department of Respiratory Diseases, Clínica Universidad de Navarra-IDISNA, Pamplona, Navarra, Spain; ^4^ Vascular and Interventional Radiology Unit, Clínica Universidad de Navarra-IDISNA, Pamplona, Navarra, Spain

**Keywords:** iung toxicity, STAT3, antiangiogenics, radioembolization, hepatocellular carcinoma

## Abstract

Sorafenib is a multi-kinase inhibitor and a vascular endothelial growth factor (VEGF) inhibitor approved to treat patients with advanced hepatocellular carcinoma, renal cell carcinoma and differentiated thyroid carcinoma. Its most common side effects are asthenia/fatigue, skin toxicity, diarrhea and arterial hypertension. Reported respiratory adverse reactions include dyspnea, cough, pleural effusion and hoarseness. The aim of this report is to describe for the first time the occurrence of pneumatocele in two patients treated with Sorafenib. Patients had no respiratory symptoms and alternative diagnoses were ruled out. Primary tumors were different (liver metastases from a pancreatic neuroendocrine tumor and hepatocellular carcinoma) but both patients had been treated with yttrium 90 radioembolization 9 and 17 months before starting on Sorafenib, respectively. No complications occurred and Sorafenib withdrawal was followed by radiologic improvement.

## INTRODUCTION

Pneumatocele is a thin-walled lung cavity filled with air usually seen as a complication of acute pneumonia [1], even though it could also arise from other noninfectious etiologies [2]. The precise pathophysiological mechanism of pneumatocele formation remains unclear although there are different advanced theories. Some suggest that the mechanism would be a combination of parenchymal necrosis and check-valve bronchiolar obstruction [3], whereas others believe in local collections of air in the interstitial tissue [4]. We present for the first time two cases of patients who developed pneumatocele while they were receiving the multikinase inhibitor Sorafenib.

## CASE PRESENTATION

### Case 1

**Figure 1:**
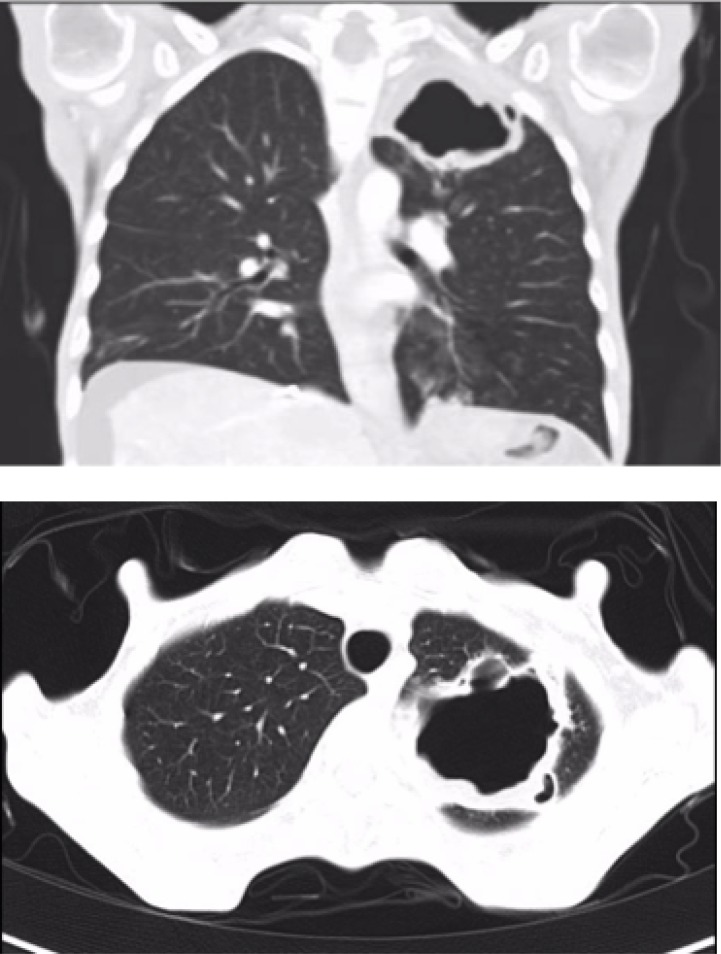
Pneumatocele in the upper left lobe (55 × 38 × 44 mm) (Case 1)

A 36-year-old woman had an invasive pancreatic neuroendocrine tumor removed by means of body and distal pancreatectomy, splenectomy, partial gastrectomy, and transversal colectomy. One-year later, multiple liver metastases were detected in both lobes. At that moment, she had chronic malnutrition despite adequate pancreatic enzyme supplementation and a Trousseau syndrome was also diagnosed. Chronic malnutrition was attributed to a combination of pancreatic insufficiency and a potential hormone-related paraneoplastic syndrome. Systemic chemotherapy with 5-fluorouracil and streptozotocin followed by transarterial bland embolization were performed, and an objective durable remission was achieved. Seven years later, liver progression was detected, restricted mostly to the right lobe. Her performance status was ECOG 1 but she had a BMI of 13.7 kg/m^2^ due to worsened chronic malnutrition. A left pleural effusion was also observed but no malignant cells were detected on fluid cytology. A right-lobe transarterial radioembolization (TARE) was uneventfully performed. The lung shunt fraction in the macroaggregated albumin scan performed during the TARE workup was 6.4%. The response to TARE was short lived and 9 months later liver disease progressed. The multidisciplinary team recommendation was to start Sorafenib as a special indication under informed consent instead of Sunitinib due to her profound asthenia. The dose used was progressively increased from 400 mg to 600 mg daily with good tolerability and no side effects. Two months later a thoracic CT showed a small pleural effusion in left side and ground glass in left lower lobe. Four months after initiating Sorafenib an abdominal and thoracic CT showed a lung cavity in the left upper lobe (Figure 1). The patient had no respiratory symptoms.

**Figure 2:**
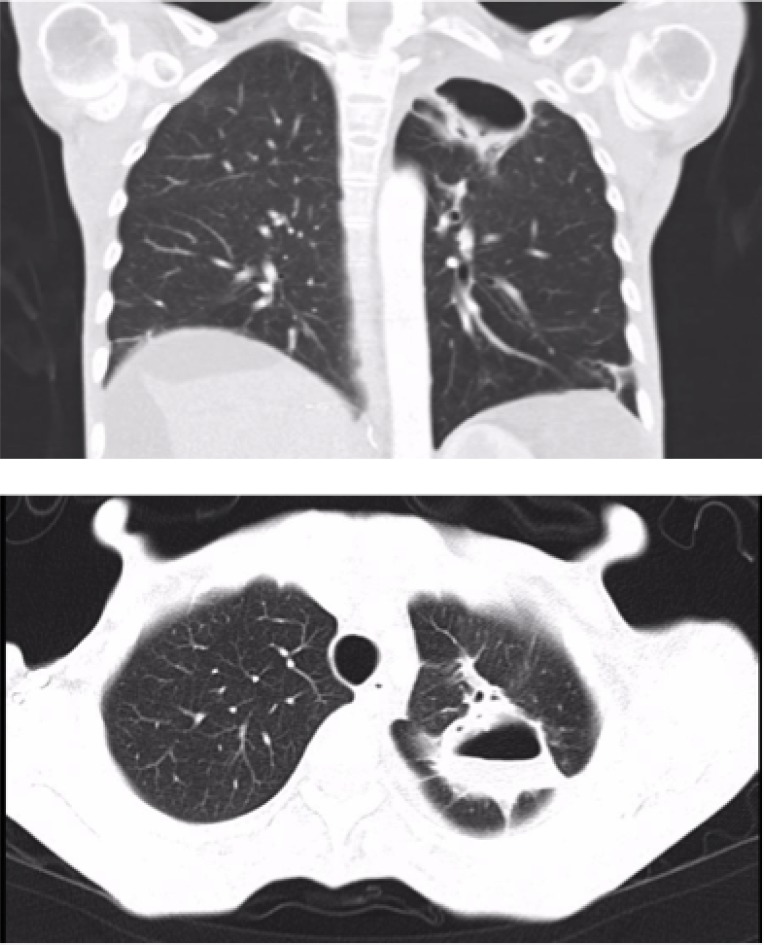
Lung cavity improvement after discontinuing Sorafenib treatment (27 × 28 × 42 mm) (Case 1)

A bronchoscopy was performed with no macroscopic findings. No malignant cells were observed on cytology of a bronchioalveolar lavage (BAL) and microbiologic cultures were negative. Sorafenib was discontinued after 4 months of treatment. Repeated CT scans performed one and three month after discontinuing Sorafenib showed a reduction in the size of the pneumatocele (Figure 2). Sorafenib was not reintroduced and the patient died 4 years after the pneumatocele was diagnosed due to tumor progression.

### Case 2

**Figure 3:**
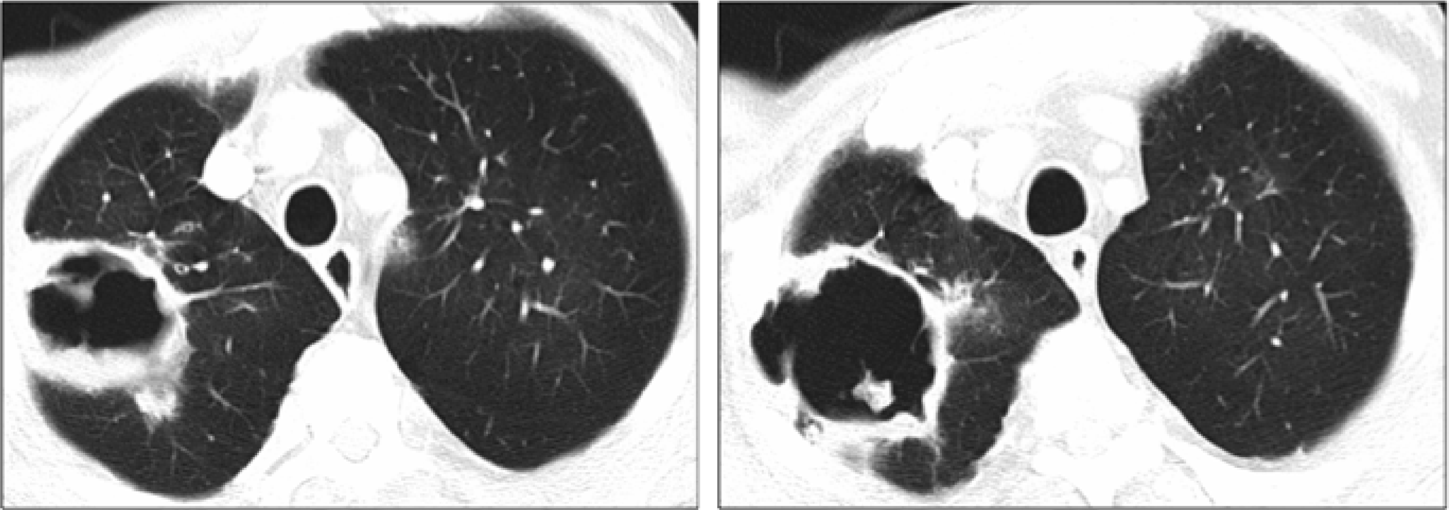
Lung cavity at diagnosis in the right upper lobe (120 × 60 × 60 mm) (Case 2)

A 62-year-old man was incidentally diagnosed of a hepatocellular carcinoma in the advanced stage due to branch portal vein invasion, as well as alcohol-related cirrhosis (Child Pugh A5). His medical history included a diagnosis of severe chronic obstruction pulmonary disease (COPD), arterial hypertension and peripheral arteriopathy. As part of a lung cancer screening program, he had a chest CT-scan performed a year before receiving Sorafenib which showed mild paraseptal emphysema in upper and lower lobes with no bullae. Selective TARE was followed by an objective prolonged tumor response. One year after TARE, a total body CT showed no signs of extrahepatic disease. Five months later, he reported abdominal pain and a liver MRI revealed liver progression. Sorafenib was started at 800 mg daily but later reduced to 400 mg daily due to intense asthenia and weight loss. The patient had usually two COPD exacerbations per year. Exacerbations were more frequent after the start of Sorafenib and he required antibiotics and systemic corticosteroids. One year after Sorafenib was initiated, he developed cough and progressive dyspnea and a chest CT scan showed a pneumatocele (Figure 3). Sorafenib was discontinued and amoxicillin-clavulanic acid was prescribed with symptom relief. Weeks later, an *Aspergillus fumigatus* was isolated in a sputum culture and oral voriconazol was introduced despite the good response to antibiotics. A new CT one month after discontinuation of Sorafenib showed a slight reduction in the size of the pneumatocele. Sorafenib was reintroduced at the same reduced dose.

**Figure 4:**
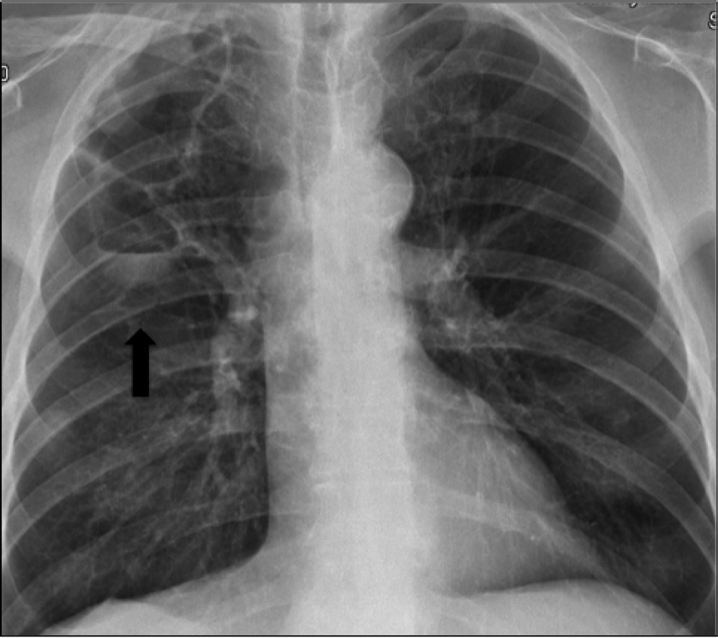
Pneumatocele enlargement detected in chest X-ray *(black arrow)* (Case 2)

Three months after restarting Sorafenib treatment, he again had asthenia, cough and purulent sputum. A chest X-ray showed an enlarged pneumatocele (Figure 4). Empiric antibiotic therapy with clindamicyn and voriconazol was prescribed. *Aspergillus fumigatus* was again isolated in the sputum but not in the BAL culture. Symptoms improved although the size of the cavity remained stable. Sorafenib was permanently discontinued after 14 months, recessed and the patient died one month later because of tumor progression.

## DISCUSSION

Pneumatocele is an air-filled cyst that develops within the lung parenchyma usually related to a respiratory infection but it could also develop in another context such as trauma or mechanical ventilation [1]. It is asymptomatic in most cases, and does not require surgical or percutaneous intervention [5]. Complications may occur including tension pneumatocele [6], pneumothorax and superinfection. These two cases share a medical history of uneventful treatment with TARE followed months later by treatment with Sorafenib. TARE may rarely produce radiation pneumonitis, with a diffuse alveolar pattern and fibrosis [7] but cavitation has not been described. On the other hand, respiratory complications are uncommon during Sorafenib therapy but include pneumonia [8] and interstitial pneumonitis [9]. To our knowledge, this is the first time that pneumatocele is reported in Sorafenib-treated patients. Although a potential role of COPD and bacterial and fungal superinfection may not be ruled out in the second patient, the similarity to the first case and the enlargement of the cavity when Sorafenib was resumed in the second case speak against such possibility.

Although unraveling the pathogenesis of pneumatocele in these two cases is beyond our prospects, we hypothesize that it might be related to the ability of Sorafenib to interfere in the human signal transducer and activator of transcription (STAT-3) pathway. STAT-3 plays a significant role in regulation of genes involved in tumor cell proliferation, survival and invasion [10]. Sorafenib decreases STAT-3 phosphorylation at both tyrosine and serine residues, regardless of Janus Kinase 2 (JAK 2) and phosphatase shatterproof 2 (SHP2) activity [11]. STAT-3 dephosphorylation induced by Sorafenib has been described in *in vitro* studies of human medulloblastomas [12] and esophageal adenocarcinomas, [13] and *in vivo* study of cholangiocarcinomas [14].

On the other hand, STAT-3 is expressed in numerous cells related to development of postnatal lung. *In vitro* studies have demonstrated a relationship between STAT-3 and surfactant protein B expression [15]. In the absence of STAT-3, mice exposed to 95% oxygen develop alterations in lung structure, permeability and surfactant, as well as lung mechanics [16]. A connection between STAT-3 and pneumatocele was described in 2007 when a STAT-3 mutation causing STAT-3 deficiency was linked to the hyper-immunoglobulin E syndrome [17], which is characterized by eosinophilia, increased IgE levels and an inadequate destructive inflammatory response in the skin and lung leading to pustular and eczematoid rashes, pneumatocele and bronchiectasis [18]. Therefore, we hypothesize that inhibition of STAT-3 by Sorafenib may lead to pneumatocele formation. An alternative explanation could be based on the antiangiogenic activity of Sorafenib acting on the lung vasculature subclinically damaged by radiation delivered by TARE, but this is even more speculative. Importantly, none of our patients smoked cannabis.

In summary, pneumatocele may be a rare pulmonary complication of Sorafenib treatment in patients previously treated by TARE. Treatment discontinuation should be considered in the context of tumor response, additional side effects and therapeutic alternatives.
